# Von der Nase ins Ohr: Feldkanzerisierung über die Tuba auditiva

**DOI:** 10.1007/s00106-022-01221-6

**Published:** 2022-08-29

**Authors:** Veronika Flockerzi, Bernhard Schick, Alessandro Bozzato

**Affiliations:** Klinik für Hals-Nasen-Ohrenheilkunde, Universitätsklinikum des Saarlandes UKS, Kirrberger Str., Gebäude 6, 66421 Homburg, Deutschland

**Keywords:** Invertiertes Papillom, Mittelohrkarzinom, Glandula parotis, Rezidiv, Bestrahlung, Inverted papilloma, Middle ear carcinoma, Parotid gland, Recurrence, Radiation therapy

## Abstract

Wir berichten über das metachrone Auftreten eines invertierten Papilloms im ipsilateralen Mittelohr nach Resektion eines endonasalen invertierten Papilloms sowie über dessen maligne Transformation. Nach mehrfachen sanierenden Operationen sowie adjuvanter Radiochemotherapie kam es zu einem Rezidiv mit intrazerebraler Manifestation. Diese führte zum Tod der Patientin drei Jahre nach Erstvorstellung.

## Abstract (en)

We report on the metachronic appearance of an inverted papilloma in the ipsilateral middle ear after resection of an endonasal inverted papilloma and its malignant transformation. After multiple surgical interventions and adjuvant radiation and chemotherapy, there was recurrence with intracerebral growth, which ultimately led to the patient’s death 3 years after the initial presentation.

## Falldarstellung

### Anamnese

Die bei Erstvorstellung 68-jährige Patientin berichtete über blutig-putride Rhinorrhö und nasale Obstruktion links seit wenigen Wochen. Ohrenschmerzen, Hörminderung oder Otorrhö wurden verneint. Es bestanden keine internistischen Komorbiditäten. Medikamente wurden nicht regelmäßig eingenommen.

### Klinischer Befund

**Figure 1:**
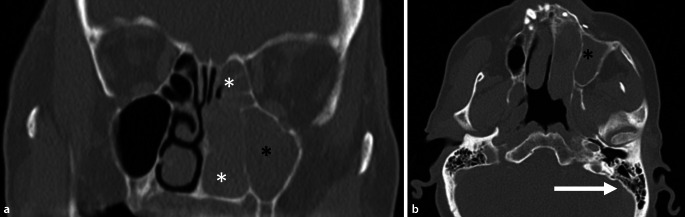

In der anterioren Rhinoskopie links zeigte sich eine polypöse, kontaktvulnerable Veränderung mit Verlegung der kompletten Nasenhaupthöhle. Die übrigen Spiegelbefunde waren unauffällig. Eine Computertomographie (CT) der Nasennebenhöhlen wurde angefertigt mit dem Befund einer linksseitigen Sinusitis unter Aussparung der Keilbeinhöhle und freier Belüftung der Mastoidzellen und des Mittelohrs (Abb. 1).

### Diagnose

**Figure 2:**
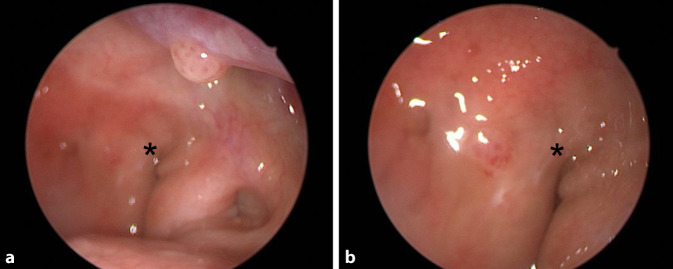

Nach Indikationsstellung zur explorierenden Nasennebenhöhlenoperation links zeigte sich eine ulzerative, kontaktvulnerable, in die Nasenhaupthöhle links wachsende Raumforderung, ausgehend vom vorderen Siebbein. Diese wurde intraoperativ als malignitätsverdächtig eingestuft. Im Schnellschnitt wurde der Verdacht auf ein invertiertes Papillom geäußert. Zugunsten einer geplanten Rhinoskopie mit Rebiopsie nach 12 Wochen wurde zunächst auf den Einsatz des Sinusbohrers verzichtet. Intraoperativ auffällig war ein weites Tubenostium [6] im Nasenrachen (Abb. 2). Die Patientin berichtete acht Wochen postoperativ über eine subjektive Hörminderung links. Bei vermutetem Paukenerguss wurde die Indikation zur Parazentese gestellt. Hier zeigte sich eine polypöse Raumforderung im Mittelohr. In der anschließend durchgeführten Computertomographie des Felsenbeins zeigte sich eine vollständige Verlegung der Mastoidzellen und des Mittelohrs links. Im Rahmen einer Biopsie wurden weitere Anteile des invertierten Papilloms histologisch gesichert. Nachfolgend wurde mit dem Ziel der lokalen Sanierung eine endaurale Tympanoplastik mit Mastoidektomie und Antrotomie links durchgeführt. Bei histologisch schweren Dysplasien im gewonnenen Gewebe wurde im Rahmen der interdisziplinären Kopf-Hals-Tumorkonferenz die Indikation zur adjuvanten Radiatio gestellt, jedoch von der Patientin abgelehnt.

### Therapie und Verlauf

Sechs Wochen später zeigte sich das linksseitige Trommelfell erneut entdifferenziert und feucht. Links zeigte sich sonographisch intraparotideal eine scharf begrenzte, rundliche und echoarme, nichtvaskularisierte Raumforderung von 7 × 6 × 4 mm, die im Rahmen einer partiellen Parotidektomie exstirpiert wurde. Simultan wurden Biopsien vom Trommelfell gewonnen. Beide Gewebeproben zeigten fokal Übergänge in ein mittelgradig differenziertes nichtverhornendes Plattenepithelkarzinom, G2.

**Figure 3:**
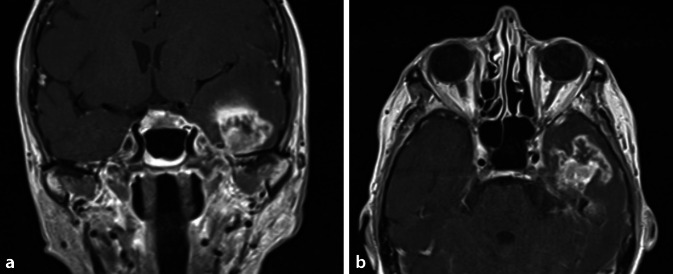

Ein erneutes CT-Staging erbrachte keinen Hinweis auf weitere Organmetastasen, es bestand jedoch der Verdacht auf ein maligne transformiertes Rezidiv des invertierten Papilloms am Septum nasi links bei rhinoskopischem Gewebsplus septal links in Regio IV. Zehn Monate nach Erstvorstellung erfolgte die operative Revision der Nase (endonasal-endoskopisch) und des Mittelohrs links. Histopathologisch wurde die Diagnose eines mittelgradig differenzierten papillären Plattenepithelkarzinoms G2 auf dem Boden des invertierten Papilloms an beiden Stellen gestellt. Im Konsens der interdisziplinären Kopf-Hals-Tumorkonferenz wurde die Indikation zur adjuvanten Radiochemotherapie gestellt und von Januar bis März 2018 appliziert (normofraktionierte Radiatio der linken Nasenhaupthöhle, der linken Kieferhöhle, des linken Gehörgangs unter Einschluss der ipsilateralen zervikalen Lymphabflusswege ad 50,4 Gy inklusive simultan integriertem Boost ad 59,92/63 Gy; simultan 6 Zyklen Chemotherapie mit Cisplatin (40 mg/qm KOF/Tag wöchentlich)). Nach initial stabilem Verlauf kam es im Mai 2019 zu einem erneuten Rezidiv des tympanalen Malignoms mit linksseitiger Fazialisparese. Die Magnetresonanztomographie zeigte eine neu aufgetretene zerebrale Raumforderung links temporal, unmittelbar angrenzend an das linke Felsenbein, vereinbar mit einer Metastase des bekannten Plattenepithelkarzinoms im Sinne einer Ausbreitung per continuitatem mit konsekutiver Mittellinienverlagerung (Abb. 3). Die Raumforderung wurde mitsamt infiltriertem Knochen durch die Kollegen der Neurochirurgie reseziert. Von HNO-Seite erfolgte die Parotidektomie links sowie eine Hypoglossus-Jump-Anastomose mit dem zervikofazialen Fazialisastanteil über die Ansa cervicalis links. Als Komplikation des neurochirurgischen Eingriffs entwickelte sich eine Sinus-cavernosus-Thrombose links. Diese wurde konservativ mit therapeutischer Antikoagulation durch niedermolekulares Heparin behandelt. Letztmalig berichtete der Ehemann im Januar 2020, seine Ehefrau wünsche keine weitere Therapie mehr, sie werde palliativ zu Hause versorgt.

## Diskussion

Das invertierte Papillom ist mit etwa 0,5–4 % aller Tumoren der Nase eine seltene Tumorentität, die beim Auftreten im Felsenbein ein höheres Entartungsrisiko (bis 60 %) aufweist als endonasal (bis 15 %) [1]. Aktuell liegen weniger als 40 kasuistische Beschreibungen eines invertierten Papilloms im Bereich des Mittelohrs vor [2–4]. Der dargestellte Fall illustriert das multilokuläre Auftreten eines maligne transformierten und zuletzt lokoregionär metastasierten invertierten Papilloms. Metastatische Absiedlungen sowie intrakranielle Ausdehnungen sind nach Kenntnisstand der Autoren in der Literatur kasuistisch [1, 2] beschrieben. Besonders herausfordernd für den Behandler sind die unspezifischen Symptome (Otorrhö, Schallleitungsschwerhörigkeit, Druckgefühl) sowie die Einschränkungen in der Radikalität der operativen Möglichkeiten aufgrund der individuellen Tumorlokalisation insbesondere bei Kontakt oder gar Durchbruch der Schädelbasis [2]. Hier ist eine interdisziplinäre Zusammenarbeit insbesondere mit der Neurochirurgie notwendig.

Das invertierte Papillom kann primär im Mittelohr entstehen, sich aber auch sekundär absiedeln. Als Ausbreitungswege werden drei Möglichkeiten diskutiert: i) per continuitatem über die Tuba auditiva; ii) im Rahmen eines multifokalen Geschehens bei versprengter Nasennebenhöhlenschleimhaut; iii) als „embolisches“ Geschehen über die Tuba auditiva, zum Beispiel im Rahmen einer Nebenhöhlenoperation [5], und iv) bei genetischer Prädisposition [2]. Im vorgestellten Fall muss bei initial beschriebenen prominenten Tubenwülsten in Erwägung gezogen werden, dass Tumorzellen über die Tuba auditiva von der Nase ins Mittelohr gelangt und dort transformiert sind. Weiterhin ist die Möglichkeit einer Feldkanzerisierung der respiratorischen Schleimhaut zu diskutieren.

## Fazit für die Praxis

- Das invertierte Papillom kann auch außerhalb des Nasennebenhöhlensystems auftreten.
- Manifestationen außerhalb des Nasennebenhöhlensystems haben ein höheres Entartungsrisiko und sollten daher gezielt behandelt und engmaschig klinisch sowie radiologisch kontrolliert werden.
- Die vorliegenden Befunde lassen vermuten, dass über die Tuba auditiva eine Verschleppung der Tumorzellen ins Mittelohr stattfand.
